# Transactions of the American Dermatological Association

**Published:** 1903-09

**Authors:** 


TRANSACTIONS OF THE AMERICAN DERMATOLOGICAL ASSOCIATION. Twenty-sixth Annual Meeting held at Boston, Mass., September 18-20, 1902. Frank Hugh Montgomery, M. D., Secretary.

This publication consists of papers read at the meeting of the association, held at Boston, in September, 1902, most of which are illustrated by photographs and appeared in the *Journal of Cutaneous and Genitourinary Diseases.* Discussions on the part of members followed each contribution.

The presidential address was made by Dr. Jackson, of New York, who went over the entire work of the organisation since its first meeting at Niagara Falls, 25 years ago. Included is a list of the officers and members. The facts involved will always be most valuable in the way of both information and reference.

The papers presented were usually confined to modern questions and discussions. Their standard is high and the aggregate constitutes a valuable addition to our current medical literature.

